# Adverse COVID-19 vaccination effects in Finnish patients with Ménière’s disease: a cross-sectional study

**DOI:** 10.12688/f1000research.113143.1

**Published:** 2022-08-04

**Authors:** Eldre Beukes, Vinaya Manchaiah, Nora Pyykkö, Ilmari Pyykkö

**Affiliations:** 1 Vision and Hearing Sciences Research Group, Anglia Ruskin University, Cambridge, Cambridgeshire, CB1 1TP, UK; 2 Virtual Hearing Lab, Virtual Hearing Lab, Collaborative Initiative between University of Colorado School of Medicine and University of Pretoria, Aurora, Colorado, USA, Aurora, Colorado, USA; 3 Department of Otolaryngology–Head and Neck Surgery, University of Colorado School of Medicine, Aurora, Colorado, USA; 4 UC Health Hearing and Balance, University of Colorado Hospital, Aurora, Colorado, USA; 5 Department of Speech-Language Pathology and Audiology, University of Pretoria, Pretoria, Gauteng, South Africa; 6 Department of Speech and Hearing, Manipal College of Health Professions, Manipal Academy of Higher Education, Manipal, India; 7 Faculty of Medicine, University of Tartu, Tartu, Estonia; 8 Department of Otolaryngology, Hearing and Balance Research Unit, University of Tampere, Tampere, Finland

**Keywords:** Ménière’s disease, COVID-19 vaccination, COVID-19, audiovestibular, vertigo, hearing loss, tinnitus

## Abstract

**Background: **The association between reporting adverse coronavirus disease 2019 (COVID-19) vaccination effects and those with a history of audiovestibular difficulties is unknown. The aim of this research is therefore to investigate adverse vaccination effects in adults with a history of Ménière’s disease. Specifically, the incidence of adverse effects, the factors associated with those reporting adverse effects and the relationship between the reporting of audiovestibular and other adverse effects.

**Methods: **A mixed-methods exploratory cross-sectional survey study design was used. Data were collected from 333 members of the Finnish Ménière Association. The survey was designed to obtain demographic information that may be associated with having adverse effects or not, vaccination-specific information and adverse vaccination effects. Both health and audiovestibular adverse events were identified. Data analysis included comparing those reporting and not reporting adverse vaccination effects.

**Results: **The mean age was 63 years with 81% being female. Of the 327 respondents who had one of the COVID-19 vaccinations (Comirnatry/ Pfizer, Astra Zeneca, or Moderna), 203 (62%) reported no adverse effects. The type of or number of vaccinations were not related to the reporting of adverse effects. The most frequently reported adverse effects were injection site tenderness (38%), arm pain (21%), fever (15%) and headaches (15%). Post-vaccination tinnitus and vertigo (both 7%) were the most frequently reported audiovestibular-related symptoms, followed by aural fullness (6%) and hearing loss (4%). Those reporting previous pre-vaccination vertigo were more likely to have post-vaccination vertigo. The presence of post-vaccination tinnitus, hearing loss, and aural fullness, predicted the presence of post-vaccination vertigo.

**Conclusions: **A small proportion of patients with a history of Ménière’s disease may experience adverse post-vaccination effects. Further research is required to explore whether adverse post-vaccination audiovestibular effects are more prevalent in those with a history of otological disorders compared with the general population.

## Introduction

The severe acute respiratory syndrome coronavirus 2 (SARS-CoV-2) infection has resulted in more than five million deaths after two years of the coronavirus disease 2019 (COVID-19) pandemic. ^1^ Many patients with COVID-19 infections fully recover, however, a proportion experience long-term symptoms including pulmonary, cardiovascular, nervous system and psychological effects. ^2^ Evidence for the association between COVID-19 infection and audiovestibular symptoms such as hearing loss, tinnitus and vertigo has furthermore been identified. ^3^^,^^4^

To reduce the risk of receiving COVID-19 infection, vaccines were developed, and global vaccination implementation recommended. ^5^ Several vaccines are approved for use and proven effective, however, post-vaccination adverse effects are also reported. ^6^ The most common reported adverse effect are pain at the site of injection, fatigue, and headache, which generally are mild and resolve within a few days. ^7^ Adverse audiovestibular effects such as sudden hearing loss, have also been reported in case studies or larger groups, such as sudden sensorineural hearing loss, ^8^^,^^9^ tinnitus, ^10^^,^^11^ and dizziness. ^12^^,^^13^ Although recovery is often reported and incidence rates appear similar to those in the general population (e.g., Formeister *et al*. ^14^; Tseng *et al*. ^15^).

As with COVID-19 infection, there may be certain populations who are more at risk for developing adverse post-vaccination effects. One group may be those with pre-existing audiovestibular problems, such as patients with Ménière’s disease who experience hearing loss, tinnitus and vertigo. A study by Wichova, Miller and Derbery ^16^ identified that 11 out of 30 patients reporting post-vaccination hearing-related symptoms had previous otologic diagnoses, including six patients with Ménière’s disease. As this possible association deserves further attention, the current study was undertaken with the aim of investigating adverse post-vaccination effects in patients with pre-existing Ménière’s Disease. The specific aims were to (i) identify the incidence of adverse effects, (ii) explore the factors associated with those reporting adverse effects, and (iii) identify the relationship between the reporting of audiovestibular and other adverse effects.

The Strengthening the Reporting of Observational Studies in Epidemiology (STROBE) guidelines for cross-sectional studies ^17^ was used to report the methods and results of the survey (see *Extended data* ^26^).

## Methods

### Study design

A mixed-methods exploratory cross-sectional survey study design was used to find out about vaccinations taken and possible side effects.

### Ethics statement

This study was conducted by the Union of Finnish Ménière Association sending a questionnaire by email to the members of the Union of Finnish Ménière Association, who had a registered email address. The Union of Finnish Ménière Association is a charitable organization consisting of eight regional Ménière Associations and the main aim of the Union is to coordinate and organize peer support for Ménière patients offered by the local associations. The Union administers centrally the above-mentioned member register, and the register adheres to the GDPR (General Data Protection Regulation) of the EU. The data collection was completely voluntary and non-invasive and therefore by Finnish law did not require formal ethical committee approval, as confirmed by the Finnish law authorities (ETENE). The study was approved on 9 September 2021 by the internal ethics board of the Union of Finnish Ménière Association and was carried out according to the principles expressed in the Declaration of Helsinki. The anonymized data set was provided to the research team.

Participants provided online written, informed consent for participation by confirming they understood how their data would be used and what the study entailed.

### Survey development and distribution

The survey questions were developed jointly by the study authors (IP, NP) and FMF (Finnish Ménière Federation). The survey included demographic questions (i.e., gender, age, and presence of vertigo prior to the vaccination), vaccination related questions (i.e., number of COVID-19 vaccinations received, type and date of the vaccinations), and adverse vaccination effects (i.e., vertigo, imbalance, drop attacks [Tumarkin’s otolithic Crisis]), hearing loss, aural fullness, tinnitus, sinus symptoms, headaches, arm pain and/or fever). Respondents were asked to report symptoms that appeared within three weeks after vaccinations. There was also the option to include descriptions and provide more information regarding the symptoms experienced. All the questions, except the descriptions were mandatory, and participants could not continue unless they answered prior questions. This may have reduced the numbers completing the full questionnaire.

The eligibility criteria included adults aged 18 years or older who provided written electronic informed consent. The survey was distributed via email to members of the FMF who were above 18 years, who were assumed to have a history of Ménière’s disease, and who had a registered email address, which accounts for 75% of their members. The survey was lunched on October 10 ^th^, 2021 and closed on October 28 ^th^, 2021. Reporting bias was minimized by allowing anonymous responses and carefully wording to be non-leading where possible.

### Data analysis

All analyses were completed in the Statistical Package for Social Sciences (SPSS) version 26.0. Significance was set to *p* ≤ .05, two-tailed. All the data was, thus using completer’s analysis. As all the questions were mandatory, there was no missing data, except for comment boxes.

Post-hoc tests were Bonferroni corrected for multiple comparisons. Continuous data are expressed as mean and standard deviation (SD). Categorical data are shown as percentages and frequencies. Initial analysis involved all the respondents (n = 333). To evaluate the vaccination effect, those who did not have the vaccination were removed (n = 6).

Subgroups were compared for those not reporting post-vaccination adverse effects and those reporting general post-vaccination effects (e.g., fever, arm pain, headaches) and those reporting vertigo-related effects (e.g., vertigo, falls, imbalance). The independent-samples *t*-test were used for continuous variables and the Chi-squared test for categorical variables together with adjusted standardized residual values during post-hoc testing. Spearman’s rho correlations (categorical variables) were used to estimate the strength of association between reporting vertigo-related problems and other post-vaccination-related effects. Correlation strength was categorized as very weak (.00 to .19) weak (.20 to .39), moderate (.40 to .59), strong (.60 to.79) and very strong (.80 to 1.0). Following this, hierarchical linear multiple regression models were performed with vertigo-related problems as the dependent variable and other post-vaccination-related effects as predictor variables. Due to the categorical data dummy variables were used. Qualitative data from the open questions were analyzed separately using inductive content analysis to supplement the quantitative analyses.

## Results

### Participant profile

**Figure 1.**
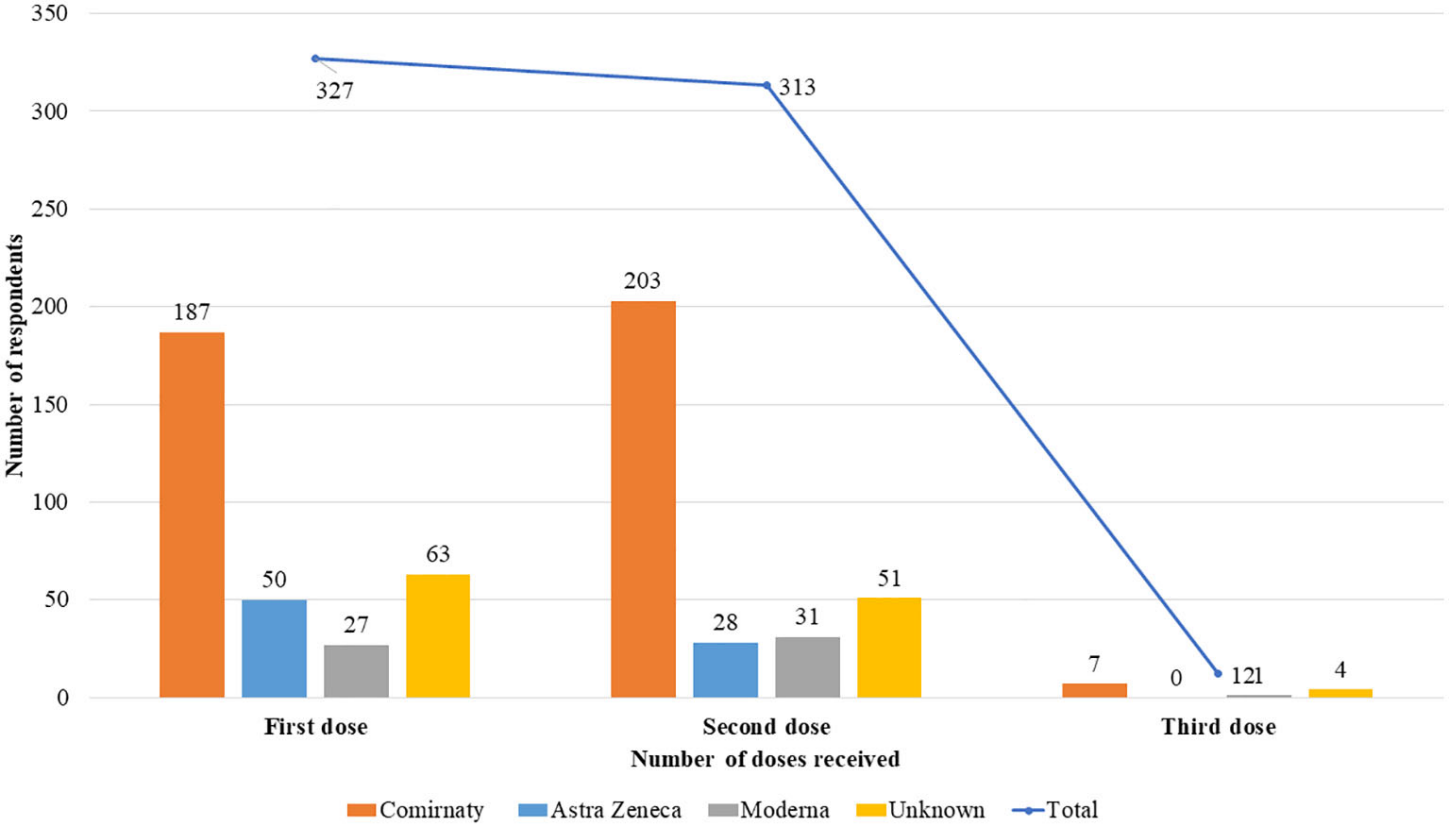
Number and type of vaccinations received.

A total of 333 participants responded to the survey from an estimated 550 participants (60% response rate). The mean age was 63 years (SD: 11 years) with an unequal gender divide with 81% being female and 19% being male as seen in Table 1, partly representing the higher incidence of Ménière’s Disease in women. ^18^ There were 31% reporting no history of vertigo prior to the vaccination and 69% reporting either constant (6%), episodic (47%) or a mixture of constant and episodic vertigo (16%). Those reporting no vertigo had a higher mean age (65 SD: 10 years) than those reporting constant (63 SD: 13 years) or episodic vertigo (62 SD: 12 years) or a mixture of constant and episodic vertigo (60 SD: 11 years). This may represent the progression of the Ménière’s disease that those that are older were in the later stages and hence having fewer symptoms. This indicated a significant relationship between age and type of vertigo reported ( *r* = -.19, *p* = 0.002). Of those responding, 327 (98%) had the first, 313 (94%) had the second and 12 (4%) had the third vaccination as seen in Figure 1. The majority were vaccinated with Comirnaty (Pfizer/BioNTech) (69%) followed by Oxford- Astra Zeneca (15%) and then Moderna (8%) vaccinations.

**Table 1.** Demographic profile of the respondents and comparison of those with and without general vaccination effects.

	All respondents (n = 333)	No vaccination effects (n = 209 in total and 203 who had the vaccination)	Effects from the vaccination (n = 124)	Between group associations/comparison
**Gender**				
Female	268 (81%)	179 (86%)	89 (72%)	χ ^2^ = 10(1), *p* < .001
Male	65 (19%)	29 (14%)	35 (28%)	
**Age (in years)**				
Mean (SD)	63 (11)	61 (11)	66 (10)	*t* = 14(332), *p* < .001
Range	27 to 89	27 to 85	32 to 89	
**Pre-vaccination vertigo experienced**				
None	102 (31%)	60 (29%)	42 (34%)	χ ^2^ = 1.6(3), *p* = .66
Constant vertigo	20 (6%)	11 (5%)	9 (7%)	
Episodic vertigo	155 (47%)	191 (49%)	54 (44%)	
Mixture of constant and episodic vertigo	53 (16%)	34 (17%)	19 (15%)	
**Number having the first vaccination**				χ *^2^* = 3.6(1), *p* = .08
Received	326 (98%)	202 (97%)	124 (100%)	Post-hoc testing depending on vaccination type: χ *^2^* = 7.6(3), *p* = .06
Not received	6 (2%)	6 (3%)	0	
**Month of the first vaccination**				χ ^2^ = .5(2), *p* = .77
Jan-March 2021	80 (24%)	51 (24%)	29 (23%)	
April-June 2021	146 (44%)	99 (47%)	47 (38%)	
July-September 2021	17 (5%)	12 (6%)	5 (4%)	
Unknown	90 (27%)	47 (23%)	43 (35%)	
**Number having the second vaccination**				χ *^2^* = 1.4(1), *p* = .18
Received	313 (94%)	194 (93%)	119 (96%)	Post-hoc testing depending on vaccination type: χ ^2^ = 4.9(3), *p* = .18
Not received	20 (6%)	15 (7%)	5 (4%)	
**Month of the second vaccination**				
Jan-March 2021	10 (3%)	9 (4%)	1 (1%)	χ ^2^ = 7(2), *p* = .02
April-June 2021	100 (30%)	55 (26%)	45 (36%)	
July-October 2021	168 (51%)	114 (55%)	54 (44%)	
Unknown	55 (16%)	31 (15%)	24 (19%)	
**Number having the third vaccination**				χ ^2^ = 2.25(1), *p* = .13
Received	12 (4%)	10 (5%)	2 (2%)	Post-hoc testing depending on vaccination type: χ ^2^ = 2.2(2), *p* = .33
Not received	321 (96%)	199 (95%)	122 (98%)	

### General vaccination effects

**Figure 2.**
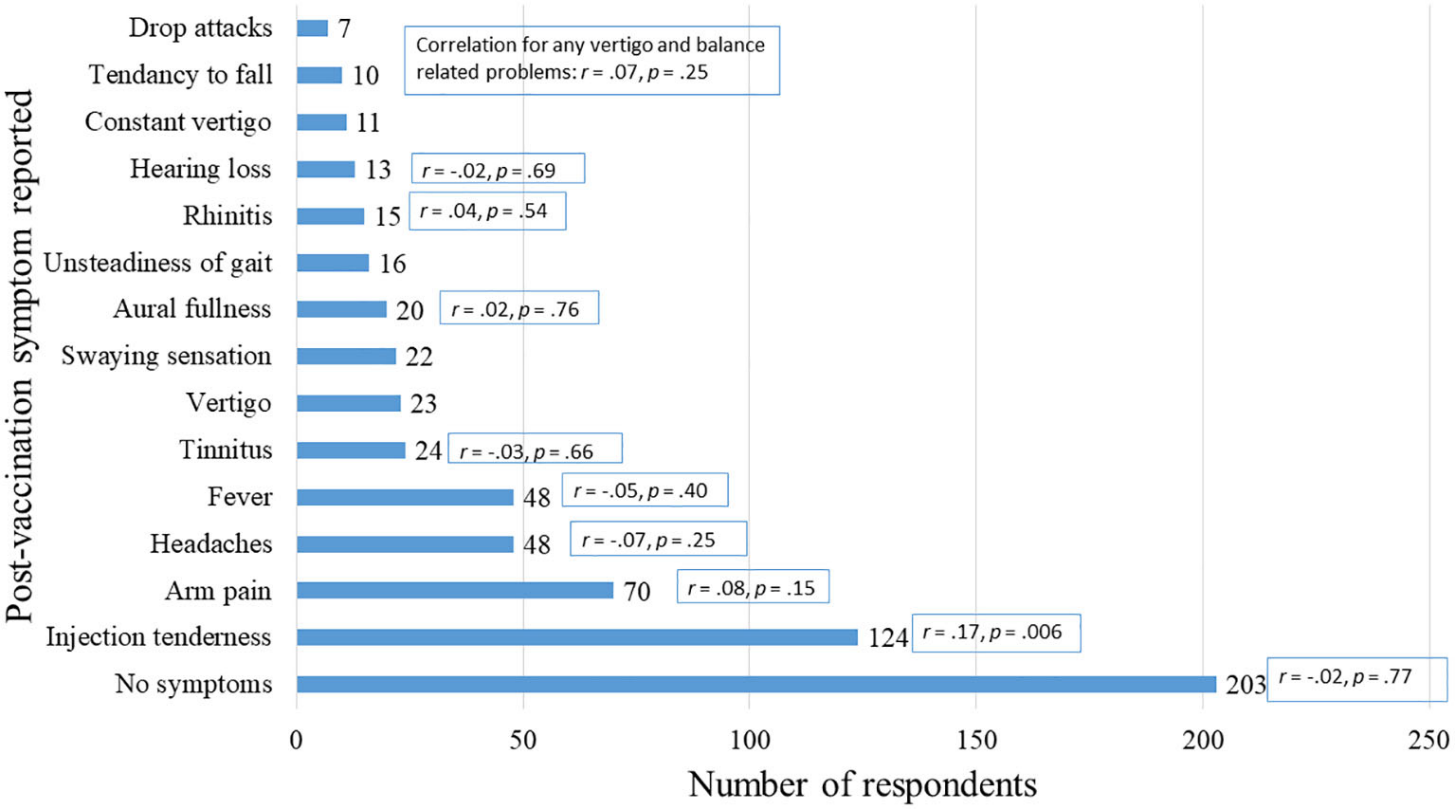
The presence of post-vaccination adverse effects. Correlation coefficients provided represent Spearman’s correlations between the symptom and type of vaccination received.

Of the 327 who were vaccinated, 203 (62%) reported no vaccination adverse effects and 124 (38%) reported post-vaccination adverse effects. The most common adverse effect was injection site tenderness (38%), followed by arm pain (21%), fever (15%) and headaches (15%) as seen in Figure 2. Although differences in symptom reporting was observed, such as less fever reported by those with Comirnaty (10%) compared to those Astra Zeneca (30%) and Moderna (33%) no significant correlations were found between the symptom reported and type of vaccination received. From the open-ended responses (see supplementary information S2) most of these symptoms were short lived, expected and mild as described by these example responses: “Mild pain at the injection site that disappeared the next day,” “Mild fever the next day after the first vaccination,” and “A headache that lasted 1 day.” Post-vaccination tinnitus and vertigo (both 7%) were the most frequently reported audiovestibular-related symptoms, followed by aural fullness (6%) and hearing loss (4%).

Table 1 compares those reporting and not reporting general post-vaccination adverse effects. There were no significant associations regarding those having effects or not, based on the type of vaccination received. Age differences were present as those having vaccination effects were significantly older. Gender differences were also seen as significantly fewer females (33%) had adverse effects compared with those with no effect (67%). Significantly more males (55%) had effects compared to those with no effects (45%). The time of vaccination had no effect, except for the second vaccination, significantly fewer respondents vaccinated in April-June experienced adverse effects (55%) compared with those without effects in this period (45%). Spearman’s correlations investigated the effect of the vaccination type and the presence of symptoms. The only weak positive correlation was for local arm pain being higher for those with the Moderna injection (although this is a smaller sample: 10 not having pain and 17 having pain). Those receiving Astra Zeneca and Moderna were less likely to report injection pain.

### Vaccination effects on vertigo and balance

Of the 327 who were vaccinated, 281 (86%) reported no vertigo, imbalance, swaying or drop attacks and 46 (14%) reported at least one vertigo-related post-vaccination symptom as seen in Table 2. Of these 23 (7%) reported one symptom and 23 (7%) reported two or more symptoms. There were no gender effects and no differences found related to the type of vaccination received. Those who were younger were significantly more likely to report vertigo and imbalance post-vaccination. There were also significant differences based on the presence of pre-vaccination vertigo-related problems as those reporting previous pre-vaccination vertigo were more likely to have post-vaccination vertigo. The strength of this relationship was a small positive relationship ( *r* = .16, *p* = .005). When looking at the group reporting vertigo as the largest vestibular complaint (n = 23), there were no significant difference between this group and those not reporting vertigo as seen in the last column of Table 2.

**Table 2.** Comparison of those reporting post-vaccination vertigo-related symptoms to those not reporting these symptoms.

	No vertigo (n=281, 86%)	Constant vertigo, episodic vertigo, falls and/or imbalance reported (n=46; 14% of this sample)	Those reporting episodic vertigo only (n=23)	Between group comparison: no vertigo and imbalance-related problems post-vaccination	Between group comparison: no vertigo and vertigo-only post-vaccination
**Gender**					
Female	224 (80%)	41 (89%)	22 (96%)	χ ^2^ = 2.17(1), *p* = .10	χ ^2^ = 3.36(1), *p* = .07
Male	56 (20%)	5 (11%)	1 (4%)		
**Age in years:** mean (SD)	63 (11)	59 9)	62 (9)	Mean difference: 4.08 years	Mean difference: 1.0 years
Range	27 to 89	41 to 80	47 to 80	*t* = 2.54(63.23), *p* = .013	*t* = .49(26.74), *p* = .63
**Pre-vaccination vertigo experienced**				χ ^2^ = 11.08(3), *p* = .01	χ ^2^ = 3.36(1), *p* = .07
None	96 (34%)	6 (13%)	5 (22%)		
Constant vertigo	15 (5%)	5 (11%)	1 (4%)		
Episodic vertigo	130 (46%)	23 (50%)	10 (44%)		
Mixture of continuous and episodic vertigo	40 (14%)	12 (26%)	7 (30%)		
**Type of first vaccination**				χ ^2^ = 2.31(2), *p* = .32	χ ^2^ = 1.59(2), *p* = .66
Asta Zeneca	46 (16%)	4 (9%)	2 (9%)		
Comirnaty	156 (56%)	31 (67%)	15 (65%)		
Moderna	23 (8%)	4 (9%)	1 (4%)		
Unknown	56 (20%)	7 (15%)	5 (22%)		
**Vertigo-related** **Symptoms**	N/A	23 (50%)	23 (100%)		
Constant vertigo		11(24%)	8 (35%)		
Episodic vertigo		22 (48%)	8 (35%)		
Swaying		10 (22%)	6 (26%)		
Tendency to fall		16 (34%)	8 (35%)		
Unsteadiness Vestibular drop attacks		7 (15%)	3 (13%)		
**Number of vertigo-related symptoms**					
1 symptom		23(50%)	9 (39%)		
2 symptoms		13 (28%)	5 (22%)		
3 symptoms		3 (7%)	3 (13%)		
4 symptoms		4 (9%)	3 (13%)		
5 symptoms		2 (4%)	2 (9%)		
6 symptoms		1 (2%)	1 (4%)		

### Association between post-vaccination vertigo and other post-vaccination adverse effects

There was a positive relationship between experiencing post-vaccination vertigo and experiencing post-vaccination tinnitus, hearing loss, aural fullness, headaches, and rhinitis as seen in Table 3. The correlation was strong for tinnitus, moderate for hearing loss and aural fullness and weak for headaches and rhinitis. Hierarchical linear multiple regression analysis was carried out to investigate the ability of the presence of these adverse effects to predict the presence of post-vaccination vertigo (see Table 3). All the available effects were used in the model. The model significantly improved the ability to post-vaccination vertigo and imbalance [ *F*(8, 318) = 34.21, *p* = 0.001*] and 46% (Adjusted *R ^2^* = 0.63) of the variance was explained by the presence of these symptoms. Removing the non-significant variables did not improve the model. The variables making significant predictors regarding the presence of post-vaccination vertigo and imbalance were tinnitus ( *β* = .50, *p* < 0.001), aural fullness ( *β* = .19, *p* < 0.001), rhinitis ( *β* = .14, *p* = 0.003) as seen in Table 3.

**Table 3.** Hierarchical linear multiple regression model with post-vaccination vertigo-related symptoms as the dependent variable and other post-vaccination adverse effects (e.g., tinnitus, hearing loss, headaches) as predictor variables. Significant results are represented by *p* = 0.05.

Variable	Spearman Correlation coefficient	Unstandardized Coefficient b (the individual contribution of each predictor to the model), CI	Coefficient standard error indicating the extent these values vary across each sample SE b	Standardized coefficients *β*	Whether the predictor is making a significant contribution to the model *t*-value ( *p*-value significance)
**Constant**		.6 [.02 to .10]			*t* = 2.94, *p* = 0.004
**Tinnitus**	*r* = -.63, *p* < .001	.68 [.54 to .80]	.07	.50	*t* = 10.62, *p* < 0.001
**Hearing loss**	*r* = -.42, *p* < .001	.03 [-.14 to .19]	.08	.02	*t* = .33, *p* = 0.74
**Aural fullness**	*r* = .50, *p* = .001	.26 [.11 to .42]	.08	.19	*t* = 3.37, *p* < 0.001
**Headache**	*r* = -.20, *p* < .001	.24 [-.06 to .11]	.05	.02	*t* = 3.37, *p* < 0.001
**Rhinitis**	*r* = .29, *p* < .001	.22 [.08 to .37]	.07	.14	*t* = 3.02, *p* < 0.003
**Local injection pain**	*r* = .10, *p* = .08	.03 [-.03 to .09]	.07	.14	*t* = 3.02, *p* = 0.003
**Arm pain**	*r* = -.02, *p* = .74	-.03 [-.10 to .05]	.04	-.03	*t* = -.72, *p* = 0.47
**Fever**	*r* = -.06, *p* = .31	-.04 [-.12 to .05]	.04	-.04	*t* = -.89, *p* = 0.38

### Onset, duration and vaccination dosage effects

From the open-ended responses it was very difficult to identify if some participants were describing vaccination-related effects or general pre-vaccination effects due to vague comments such as “Hearing problems in my right ear,” Constant feeling of pressure,” or “headaches.” A summary of the number of open-ended responses for each symptom and information regarding the onset, duration and vaccination dosage where available is available in *Underlying data.* The onset of vertigo and unsteadiness was between 12 hours to two and a half weeks post-vaccination. Where reported, the duration was between a few hours to two weeks. Effects were reported for either of the vaccination doses and at times both the first and second doses. Some patients reported that they felt that their symptoms were exacerbated by the stress during the pandemic and not necessarily the vaccination.

## Discussion

The aim of the current study was to investigate adverse post-vaccination effects for 333 Finnish patients with an assumed history of Ménière’s disease due to recruitment through the FMF. Of the 327 who were vaccinated, 203 (62%) reported no adverse vaccination effects and 124 (38%) reported one or more adverse effect. The most common effect was injection site tenderness (38%), followed by arm pain (21%), fever (15%) and headaches (15%). Those reporting effects were more likely to be older or to be males. This is in contrast to adverse vaccination effect being higher in the female population in the general public as reported in previous studies. ^19^ It may be that the gender imbalance of the sample size is affecting these results and they should thus be interpreted with caution. Some vaccination reports have found no age effects ^20^ whereas others have found that young age was correlated with more effects. ^21^ For the second vaccination, significantly fewer effects were reported for those vaccinated in April-June 2020. When comparing those who reported adverse effects and those who did not, there were no significant differences base on the type of vaccination received.

Of those vaccinated, 15% reported at least one adverse audiovestibular symptom. Post-vaccination tinnitus and vertigo (both 7%) were the most frequently reported audio-vestibular symptoms, followed by aural fullness (6%) and hearing loss (4%). There were no gender or vaccination type effects but those with previous vertigo problems and younger adults were more likely to report vertigo-related problems. From the reports the symptoms appeared to resolve within two weeks of onset, although not all participants reported the duration of the effects. There was also no clear pattern as to which vaccination could result in more effects and for one group of people it was one vaccination while others reported adverse effects after both the second and third vaccinations. These results are different to those reported by Wichova *et al*. ^16^ who found that 30 (3%) of their sample of 1,325 patients reported audiovestibular effects, with hearing loss (83%) being most frequently reported, followed by tinnitus (50%), dizziness (27%) and vertigo (17%) although evidence of a correlation was not found. A further interesting difference was that the onset of audiovestibular problems was 10 days post-vaccination, which appears similar to the present study reporting onset between 12 hours to 2.5 weeks post-vaccination. Both studies suggested that previous otologic diagnoses may result in a higher incidence of post-vaccination adverse effects. A further study by Ciorba *et al*. ^22^ reported a higher incidence of post-vaccination vertigo (.96%) compared with tinnitus (.11%) in Italy. Formeister *et al*. ^14^ found that the incidence of sudden sensorineural hearing loss was similar post-vaccination to that expected in the general population. Further systematic reviews are required to identify wider audiovestibular adverse post-vaccination effects due to these difference across studies.

In the present study, experiencing post-vaccination tinnitus, hearing loss, and aural fullness predicted the presence of post-vaccination vertigo (explaining 46% of the variability). This indicates that likelihood that post-vaccination vertigo is more likely in the presence of other post-vaccination effects. The effect of stress during the pandemic was noted by numerous participants as contributing to their audiovestibular problems as previously found. ^23^ Ensuring support for such individuals is available, is required.

Overall, the current exploratory study has highlighted that a small proportion of patients with a history of Ménière’s disease may experience adverse post-vaccination effects. These individuals may be more hesitant to undergo vaccinations, particularly if they had an adverse effect for one of the vaccination dosages. Further robust studies to explore this effect is required, together with systematic reviews to pool what is known regarding post-vaccination audiovestibular effects. Further research is also required to explore whether adverse post-vaccination audiovestibular effects are more prevalent in those with a history of otological disorders compared with the general population.

### Study limitations

There were numerous limitations that should be considered when interpreting the results. Firstly, there is possible sampling bias as those responding to the survey may be patients more likely to have had post-vaccination effects. The sample was not well balanced due to an unequal gender divide, which may have affected results, although Ménière’s disease is known to be more prevalent in females. ^18^ The survey could have been improved to ask specific questions regarding the onset, duration and dosage linked to the adverse effects. Vertigo-related problems are also frequently experienced during cardiac problems. Post-vaccination vertigo could be associated with cardiovascular problems ^24^ and other non-auditory health conditions this association should be accounted for in future studies. Looking at the impact of comorbid health conditions on adverse vaccination effects is also required. Associations between other health conditions and audiovestibular symptom have been previously reported. Pyykkö *et al*. ^25^ for instance identified that vestibular syncope (sudden and transient loss of consciousness) was associated with Tumarkin attacks, migraine and history of ischemic heart disease and history of cerebrovascular disease). It is also important to establish if there are any associations regarding previous COVID-19 infections and adverse vaccination effects. Further studies and systematic reviews are encouraged to identify the incidence and mechanisms of adverse audiovestibular vaccination effects.

## Data availability

### Underlying data

Figshare: COVID 19 vaccine in Ménière's disease, https://doi.org/10.6084/m9.figshare.19519801. ^26^

This project contains the following underlying data:

- COVID vaccine in MD for repository.xslx (raw data).

### Extended data

Figshare: COVID 19 vaccine in Ménière's disease, https://doi.org/10.6084/m9.figshare.19519801. ^26^

This project contains the following extended data:

- Finnish_MD COVID Vaccination Questionnaire
- English_MD COVID Vaccination Questionnaire
- S 1S1 The STROBE Checklist

Data are available under the terms of the Creative Commons Attribution 4.0 International license (CC-BY 4.0).
