# Supplementary material for: Adverse COVID-19 vaccination effects in Finnish patients with Ménière’s disease: a cross-sectional study
Source: F1000Res. 2022 Aug 4;11:893. [Version 1] doi: 10.12688/f1000research.113143.1 (PMC9706144; doi:10.12688/f1000research.113143.1)
Supplement: Peer review history

# Peer review: Reviewer response for version 1

**DOI:** 10.5256/f1000research.124873.r153973

**Published:** 2022-11-28

**Authors:** Sun-Uk Lee

**Affiliations:** 1 Department of Neurology, Korea University Medical Center, Seoul, South Korea

The authors investigated the adverse reaction of COVID-19 vaccination among MD patients through a questionnaire survey. I personally read it with great interest and was convinced this report can provide a novel and robust message for neurotologists. However there are some issues that should be answered and addressed clearly.

 I believe it would be better to focus on the audiovestibular side effects. It seems more appealing considering the readership rather than reporting all the other systemic miscellaneous side effects. Otherwise, how about describing the audiovestibular side effects upfront in the result and discussion, and showing the other systemic adverse reactions in the latter section?

 In addition, the authors cannot be sure whether those side effects come specifically from the COVID-19 vaccination or just from ‘the vaccination’, since MD patients often report aggravation of their symptoms following other vaccinations, too. I understand that this issue cannot be answered given the study design, but it should be covered in the discussion at least, and acknowledged as a limitation of this study.

 Another major drawback of this study is that the authors associated the adverse reaction as a whole. Instead, the symptoms should be compared for each individual, not as a whole. Since MD has a episodic nature – not like other systemic symptoms that can be monophasic to occur just after vaccination --, the association cannot be decided unless those were compared for each individual. The fact that those with frequent vertigo spell also experience frequent adverse reactions indicates that it may be not because of the vaccination but rather due to uncontrolled MD, per se.

 Besides, due to its questionnaire-based study design, there may be heterogenous patients included, and the baseline nature of MD cannot be assessed, such as early vs. burnt-out, unilateral vs bilateral, which may give further information and in-depth discussion.

Is the work clearly and accurately presented and does it cite the current literature?

Yes

If applicable, is the statistical analysis and its interpretation appropriate?

Yes

Are all the source data underlying the results available to ensure full reproducibility?

Yes

Is the study design appropriate and is the work technically sound?

Yes

Are the conclusions drawn adequately supported by the results?

Yes

Are sufficient details of methods and analysis provided to allow replication by others?

Yes

Reviewer Expertise:

Neurotology, neuro-ophthalmology

I confirm that I have read this submission and believe that I have an appropriate level of expertise to confirm that it is of an acceptable scientific standard, however I have significant reservations, as outlined above.

# Peer review: Reviewer response for version 1

**DOI:** 10.5256/f1000research.124873.r153971

**Published:** 2022-11-14

**Authors:** Habib Georges Rizk

**Affiliations:** 1 Department of Otolaryngology, Medical University of South Carolina, Charleston, SC, USA

The study is clear and well done. There are limitations to the study and the authors acknowledge them. However the study results do give the clinician some answers to better counsel Meniere's disease patients when they ask abut the vaccination. Patients with prevaccination episodes are at higher risk to develop vertigo post vaccination

- What is the Finnish meniere federation and how is it different from union of Finnish meniere association?
- Was there a reason why the authors shows three weeks as the timeline after vaccination that they enquired about? Any literature cited that most side effects of vaccinations are typically within three weeks? If not please explain the rationale
- Why did the authors lump constant vertigo and episodic vertigo together and then look at episodic vertigo only? Did they attempt to separate them like they classified the prevaccination symptoms?
- "There were no gender or vaccination type effects but those with previous vertigo problems and younger adults were more likely to report vertigo-related problems." I don't see an odds ratio calculated with confidence intervals

Is the work clearly and accurately presented and does it cite the current literature?

Yes

If applicable, is the statistical analysis and its interpretation appropriate?

Partly

Are all the source data underlying the results available to ensure full reproducibility?

Yes

Is the study design appropriate and is the work technically sound?

Yes

Are the conclusions drawn adequately supported by the results?

Yes

Are sufficient details of methods and analysis provided to allow replication by others?

Yes

Reviewer Expertise:

Neurotology

I confirm that I have read this submission and believe that I have an appropriate level of expertise to confirm that it is of an acceptable scientific standard, however I have significant reservations, as outlined above.

# Peer review: Reviewer response for version 1

**DOI:** 10.5256/f1000research.124873.r148131

**Published:** 2022-10-27

**Authors:** Hari Prakash P

**Affiliations:** 1 Department of Speech and Hearing, Manipal College of Health Professionals, Manipal Academy of Higher Education, Manipal, Karnataka, India

The manuscript aims to explore the post vaccination adverse effect on individuals with Meniere’s disease. The authors have done a survey on Finnish population using questionnaire and contrasted the subgroup with the predominantly aural symptoms versus other symptoms. The authors found that the vertigo, tinnitus, and hearing loss were the most common symptoms post vaccination. Presence of tinnitus, hearing loss and aural fullness predicted the post vaccination vertigo. Overall, the study is well designed and executed. Statistics were appropriate. The study is important now since, Corona vaccination can have various adverse effect and individuals with Meniere’s disease could have some serious audio-vestibular consequence. The study should be appreciated for including a large sample of Meniere’s disease. Following are some minor clarifications that can be addressed to improve understanding of the paper.

1. If current medication history for group is available, it could be added to the results and see if it has an interaction with these side effects. 
2. It would be good add a justification if any for relating symptoms up to three weeks as a post vaccination adverse effect, also, some places it is given as 2.5 weeks, please clarify in the paper.
3. The English MD vaccination questionnaire has number of places where it is mentions “coronary vaccination”, it could be check whether it is a typo, or do they mean something else?
4. Rhinitis related aural symptoms like fullness, tinnitus, and hearing loss could it be confounder to infer the effects of vaccine on Meniere’s disease?
5. Is there any literature on audio vestibular symptoms in general population? If so, it is worth discussing in the discussion to compare how audio-vestibular symptoms in Meniere’s population differ from general population.
6. It would be interesting to know why the older individuals have higher chance of audio vestibular symptoms.

Is the work clearly and accurately presented and does it cite the current literature?

Yes

If applicable, is the statistical analysis and its interpretation appropriate?

Yes

Are all the source data underlying the results available to ensure full reproducibility?

Yes

Is the study design appropriate and is the work technically sound?

Yes

Are the conclusions drawn adequately supported by the results?

Yes

Are sufficient details of methods and analysis provided to allow replication by others?

Yes

Reviewer Expertise:

NA

I confirm that I have read this submission and believe that I have an appropriate level of expertise to confirm that it is of an acceptable scientific standard.
